# Dosimetric Parameters in Hypofractionated Stereotactic Radiotherapy for Brain Metastases: Do Flattening Filter-Free Beams Bring Benefits? A Preliminary Study

**DOI:** 10.3390/cancers15030678

**Published:** 2023-01-21

**Authors:** Diana M. Ghemiș, Loredana G. Marcu

**Affiliations:** 1 Faculty of Physics, West University of Timisoara, 300223 Timisoara, Romania; 2 MedEuropa, 410191 Oradea, Romania; 3 Faculty of Informatics and Science, University of Oradea, 410087 Oradea, Romania; 4 Cancer Research Institute, University of South Australia, Adelaide 5001, Australia

**Keywords:** hypofractionation, stereotactic radiotherapy, brain metastases, flattening filter-free beam

## Abstract

**Simple Summary:**

The use of flattening filter-free beams (FFF) in stereotactic radiotherapy has increased in recent years due to their dosimetric and clinical benefits. This study evaluated and compared two treatment plans (one employing FFF beams and one with optimized flattened beams) for eighteen patients treated with hypofractionated stereotactic radiotherapy for brain metastasis. Physical and dosimetric parameters were analyzed, resulting in no significant differences regarding the number of monitor units, conformity index (*p* = 0.28), dose gradient index (*p* = 0.4) and dose to normal brain tissue (*p* = 0.51). The average difference in target coverage was 1.26%, with lower doses for treatment plans where FF beams were employed (*p* = 0.03). Beam on time was significantly reduced for FFF beams (*p* ≤ 0.001).

**Abstract:**

Purpose: This study aimed to compare the dosimetric results of flattening filter-free (FFF) vs. flattened (FF) treatment plans for fractionated stereotactic radiotherapy (fSRT), with the goal to highlight potential advantages of FFF beams. Methods: A group of 18 patients with brain metastases treated with fSRT (30 Gy delivered in 5 fractions) were included. The dosimetric parameters evaluated were: (1) physical dosimetric parameters (number of monitor units (MUs), conformity index (CI), dose gradient index (DGI), beam on time (BOT)); (2) clinical dosimetric parameters pertaining to target volume (PTV) and organs at risk (OARs). Two treatment plans were performed for all patients: one used 6 MV FFF beams and the other used 6 MV flattened beams. Results: A slight increase in MUs was observed for the FFF mode (+23.3 MUs). The CI showed a difference of −2.7% for the FF plans (*p* = 0.28), correlated with a poorer coverage of the PTV. DGI values reported in terms of PTV are in line with international recommendations and showed a +1.9% difference for FFF plans. An average BOT of 90.3 s was reported for FFF plans, which was 2.3 times shorter than that required for FF plans delivery (*p* ≤ 0.001). A slight decrease of PTV coverage (−1.26%, *p* = 0.036) for FF plans can be considered relevant, but no other significant differences were observed between the two optimizations. No statistically significant benefit of using FFF beams to reduce V_20_ for normal brain could be demonstrated. Conclusion: These dosimetric results encourage the implementation of fSRT with standard flattened beams in centers where FFF linacs are not available.

## 1. Introduction

Brain metastases are associated with poor survival outcomes and are developed by 30–40% of cancer patients at some point of their disease. Most patients with brain metastases are diagnosed with primary tumors in the lung, breast or melanoma [1,2,3,4]. To date, there are several conventional therapeutic options for the treatment of brain metastasis which include surgery, whole brain radiation therapy (WBRT), stereotactic radiosurgery (SRS) or chemotherapy.

In recent years, the management of brain metastases has shifted towards SRS treatments due to improved clinical outcomes in terms of cognitive function without compromising overall survival, offered by the highly targeted dose delivery and the large dose gradient that spares normal structures [2]. The popularity gained by SRS is also due to the convenience of one large dose that decreases the overall treatment duration and increases the patient’s comfort. Studies showed preservation of cognitive function after SRS compared to WBRT (6–24% cognitive decline with SRS compared to 35–52% with WBRT) [5,6]. The evidence towards normal tissue complications after SRS is limited, with the most commonly reported toxicities being edema and radionecrosis [7].

Radiobiological considerations of tumor hypoxia and intrinsic radioresistance suggest the use of fractionated radiotherapy, which for the management of brain metastases is implemented as stereotactic radiotherapy (SRT). Unlike SRS, which implies the delivery of one single large dose, SRT (or fSRT) entails a fractionated radiotherapy regimen, though in fewer fractions (4–5) and over a shorter time period than conventionally fractionated radiotherapy. The reduced number of fractions brings advantages similar to radiosurgery while potentially increasing the therapeutic index by superior sparing of the normal tissue through avoidance (or at least limitation) of radionecrosis [8].

To facilitate the delivery of high dose gradients in stereotactic treatments, modern linear accelerators have the ability to administer the photon dose without the flattening filter. While small radiation fields flattening filter-free beams (FFF) have a number of advantages, such as higher dose rate, reduced peripheral dose and scatter, and reduced treatment time [9], the question still arises as to what overall benefits can FFF beams bring in SRT compared to the standard flattened beams (FF). This is an important issue for clinics lacking FFF linear accelerators while employing SRT techniques.

The aim of the current study was to compare the dosimetric results of FFF vs. FF treatment plans for fSRT, with the goal of highlighting the potential advantages of FFF beams and at the same time illustrating the dosimetric outcomes for the use of standard flattened beams for fSRT. Specifically, two classes of dosimetric parameters were evaluated: (1) physical dosimetric parameters (number of monitor units, conformity index, dose gradient index, beam on time); (2) clinical dosimetric parameters pertaining to PTV and OARs.

## 2. Methods and Materials

### 2.1. Patient Selection

Eighteen patients with brain metastases treated with hypofractionated stereotactic radiotherapy using an Elekta Infinity medical linear accelerator (linac) were included in this study. Thirteen patients had solitary lesions and five had two lesions with a minimum volume of the planning target volume (PTV) of 2 cm^3^ but not larger than 10 cm^3^. The median volume of PTV was 5.2 ± 1.9 cm^3^. The pathologies of the primary lesions were the following: lung cancer, breast cancer, renal carcinoma and melanoma (Table 1). The gender distribution was equal (9 men and 9 women), with a mean age of 65.8 ± 9.6 years.

### 2.2. Dose Prescription and Fractionation

Hypofractionated stereotactic radiotherapy (HSRT) was performed for all patients with a total dose of 30 Gy delivered in 5 fractions (6 Gy/fr). The dose of 30 Gy was prescribed to the 95% isodose line to achieve at least 80% coverage of the PTV. Hypofractionation was elected due to its advantage over SRS regarding normal tissue sparing. This fractionation regimen is recommended by international organizations including the American Society for Radiation Oncology (ASTRO) and was successfully implemented in our clinic.

### 2.3. Patient Set-Up and Simulation

Each patient underwent simulation using the Siemens SOMATOM RTPro Computed Tomography (Siemens Healthineers, Erlangen, Germany) with a dedicated protocol, with 1 mm slice thickness. The simulation was performed in supine position using a double-layered 3-point thermoplastic mask dedicated to stereotactic treatments for immobilization, together with the Elekta HeadSTEP iBEAM evo System. HeadSTEP iBEAM evo is a couch extension module with 23 angles and shoulder adapters which offers the flexibility of treatment planning with multiple non-coplanar beams owing to its shape.

### 2.4. Treatment Planning

The treatment planning system used for both contouring and treatment planning was the Elekta Monaco, version 5.51. In terms of target, three volumes were contoured for each case: gross target volume (GTV), clinical target volume (CTV) and planning target volume (PTV). The GTV was defined based on the simulation CT and additional images originating axial magnetic resonance imaging (MRI) datasets from T1 or T2 sequences and contrast CT scans. All datasets were imported into the TPS to assist physicians with the target delineation. All scans were performed over a short period of time prior to treatment planning (within 14 days). Diagnostic MR images have different characteristics as they were performed at various imaging centers, other than the radiotherapy clinic. All patients included in this study had MRI scans, while six patients also had contrast CT scans. All diagnostic images were imported into the TPS for target volume delineation and contouring. PTV was defined by expanding the GTV contour with 2 mm.

The isocenter was placed in the geometric center of the GTV established by the TPS. For all cases, coplanar and non-coplanar beams were used (Table 2). Non-coplanar beams bring important benefits in dose sparing together with the use of flattening filter-free beams. The employed linac was an Elekta Infinity able to deliver 6 MV and 10 MV energy flattened photon beams and 6 MV unflattened photon beams. The Elekta collimator assembly, Agility, is designed with 160 tungsten leaves of 0.5 width with a speed of 6.5 cm/s as key characteristics. The multileaf collimator (MLC) position is tracked in real time for an accurate treatment delivery using the Rubicon optic technology. All patients were treated with FFF beams, while treatment plans with FF beams were simulated for comparative purposes, to fulfill the aim of the study.

Treatment delivery was completed with partial arcs via volumetric modulated arc therapy (VMAT) technique. The arc length, couch angle and collimator angle were established by the medical physicist according to patient anatomy and tumor position in terms of organs at risk (OARs). The number of treatment fields (arcs) was directly correlated with the complexity of the plan and increases for cases where high dose sparing is needed.

For all patients, two treatment plans were performed: one using 6 MV FFF beams (1200 MU/min) and the other using 6 MV flattened beams (500 MU/min), with all other optimization functions of the TPS being kept constant.

### 2.5. Dosimetric Evaluation

For target volume evaluation, the conformity index (CI) is an important dosimetric parameter recommended by the Radiation Therapy Oncology Group (RTOG) to describe the quality of stereotactic plans. A CI value between 1.0 and 2.0 is considered in agreement with RTOG protocol, while a value higher than 2.5 or lower than 0.9 reflects major deviations [10]. The CI formula proposed by RTOG is:

(1) $${CI}_{RTOG}=\frac{V_{RI}}{TV}$$

where $V_{RI}$ is the volume encompassed by the prescription isodose and *TV* is the target volume. This index comes with one disadvantage: the lack of consideration of the spatial overlap of the target and treated volumes. The Paddick CI was proposed in 2000 with the aim of eliminating false scores than can be provided by the RTOG index method and is calculated using the formula:

(2) $${CI}_{Paddick}=\frac{{TV}_{PIV}^{2}}{TV\times V_{RI}}$$

where *TV* is the target volume, ${TV}_{PIV}^{}$ is the volume of the target that receives the prescribed dose and $V_{RI}$ is the total volume covered by the prescription isodose. Dose-volume histograms are used to determine the mentioned parameters [11,12]. Paddick’s formula is employed by the Monaco TPS in order to calculate the CI [13].

Reynolds et al. [14] proposed a new method of brain SRS and SRT plan evaluation using the dose gradient index (DGI) which takes into account the dose falloff. We use this index as an important evaluation factor for all SRS and fSRT treatments in our clinic with good dosimetric outcomes regarding treatment planning. DGI was calculated for both FFF and FF plans using the formula:

(3) $$DGI=100-\left\{100*\left(\left(R_{eff,50\%Rx}-R_{eff,Rx}\right)-0.3\;\mathrm{cm}\right)\right\}$$

where $R_{eff}$ is the effective radius of the prescribed dose and $R_{eff,50\%Rx}$ is the effective radius of the 50% isodose.

Target volumes and OARs were evaluated using local protocols, as presented in Table 3. Dose constraints used are valid for up to six fractions and take into account the prediction of acute intercerebral hemorrhage (ICH).

The volume of normal brain tissue that receives 20 Gy (V_20_) was evaluated as a predictor of radiation necrosis, which can interfere with the radiation oncologist’s decision regarding dose prescription.

### 2.6. Statistical Analysis

To analyze the differences between the treatment plans optimized with FFF beams and those optimized with FF beams, the following physical and dosimetric parameters were evaluated: number of monitor units, conformity index, dose gradient index, beam on time, coverage for GTV, CTV and PTV, maximum dose, mean dose and V_20_ for normal brain tissue. The Student’s t-test was used, with a statistically significant value of *p* established to be lower than 0.05.

## 3. Results

The following parameters were assessed in this comparative dosimetric analysis: (1) the physical dosimetric parameters (overall MUs delivered, CI, DGI, beam on time); (2) dosimetric evaluation of PTV and OARs.

### 3.1. Comparative Evaluation of the Physical Dosimetric Parameters

The mean number of monitor units delivered in the optimized treatments using FFF beams was 1261.2 MUs (range between 773.1 and 1776.7), with a standard deviation of 247.2 MUs (Table 1). A difference of only +23.3 MUs was observed compared to treatment plans optimized with standard flattened beams where the mean number of MUs is 1284.5 ± 260.3 (*p* = 0.29).

The calculated CI from TPS shows a difference of −2.7% (Table 4) for the FF treatment plans, indicating a slightly poorer coverage of the PTV in terms of prescribed dose, as can be seen in Table 5. This difference is, however, statistically and clinically insignificant (*p* = 0.28).

The ideal DGI values reported by Reynolds et al. [14] are defined in terms of PTV. For tumors with small volumes (1–3 cm^3^), the DGI value is between 72 to 81; for 3–5 cm^3^ volume ranges, the DGI varies between 65–74; and for larger volumes (5–10 cm^3^), the DGI is between 58 to 70. Four patients treated with fSRT had a PTV between 1–3 cm^2^ (2.55 ± 0.28 cm^3^), three patients had a volume between 3–5 cm^3^ (4.45 ± 0.05 cm^3^) and the majority had a tumor volume between 5–10 cm^3^ (6.39 ± 1.3 cm^3^). All evaluated treatment plans for both beam types had a DGI between the indicated limits (Table 4). Treatment plans optimized with FFF beams showed a small difference (+1.9%) for the DGI value compared to FF plans, but with no statistical significance (*p* = 0.4). The DGI is correlated with the dose falloff which takes into account the 50% isodose of the prescribed dose, in this case, 15 Gy. The dose gradient between the 15 Gy and 20 Gy isodose lines can be very small since Monaco TPS is able to achieve a dose gradient of 1 Gy over 1 mm. Therefore, a correlation can be made with the V_20_ dosimetric parameter for the healthy brain which shows a slight increase for FF plans (+2.95%) (Table 5).

Beam on time (BOT) is another parameter that was measured for each plan using the beam timer from the linac as a physical parameter. Treatment delivery time is also estimated by the TPS (Table 4), but the manufacturer states that the estimated delivery time is dependent on the complexity of the plan and may vary very slightly or be completely different from the actual delivery time of each treatment field. An actual measurement of delivery times is statistically more relevant due to the fact that the dose rate during treatment plan delivery cannot be limited in the TPS. Thus, in reality, delivery times will be extended by reducing the dose rate during treatment with flattened beams. The average BOT for FFF treatment plans was only 90.3 s compared to FF beams, which required 2.3 times longer BOT (average of 207 s) (*p* ≤ 0.001) due to a low dose rate of only 500 MU/min and a similar number of MUs for the delivered plan.

### 3.2. Comparative Evaluation of PTV and OAR Dosimetry

In terms of target volumes coverage, GTV coverage is kept almost constant for both optimizations (Table 5), while the difference is less than 1% for CTV coverage. A slight decrease of −1.26% can be seen for PTV coverage for FF treatment plans (*p* = 0.036). This difference is due to the optimization process which attempts to achieve a high dose gradient. The main characteristic of FFF beams is the central peak, which allows a high dose falloff at target edges for an increased dose gradient. For FF beams, the dose gradient is achieved by decreasing the target volume coverage. The maximum dose inside PTV was kept below 145% of the prescribed dose, with no statistically significant differences between FFF and FF beam optimizations (*p* = 0.864).

No statistically significant benefit of using FFF beams to reduce V_20_ could be demonstrated, as the overall difference between the two plans in terms of V_20_ was shown to be less than 0.2 cm^3^. This is well in line with the local guidelines, whereby V_20_ for the normal brain tissue must be kept below 10 cm^3^ to avoid radionecrosis. This limit is increased for larger tumors (V > 10 cm^3^) or in situations when multiple brain metastases are treated using a single isocenter technique.

### 3.3. Toxicities and Follow-Up

All 18 patients completed treatment without toxicity-caused interruption or signs of radiation necrosis. Patient evaluation was performed by a toxicology nurse or a radiation oncologist and reported in agreement with the Common Terminology Criteria for Adverse Events (CTCAE). Each patient was evaluated at least once during treatment for neurological symptoms, but no major events were reported. Grade 1 asthenia, nausea and headache were reported, but no grade 2 or higher toxicities were reported.

The short-term follow-up (3 months) period is not adequate for the evaluation of relevant signs of radionecrosis. A long-term follow-up is recommended for an adequate evaluation of radionecrosis and cognitive functions. Since stereotactic treatments have only recently been implemented in our clinic, long-term follow-up evaluation (9–12 months) is not feasible at the time of reporting.

## 4. Discussion

The dose prescription of 30 Gy delivered in 5 fractions presented in this study is an alternative to radiosurgery recommended by guidelines based on clinical trials, with the aim of reducing the volume of normal brain tissue irradiated with high doses. Normal brain tissue is considered a late-responding tissue, with a low α/β ratio. The main concern when treating brain metastases is the probability of radiation-induced toxicities, more specifically radiation necrosis [15]. To minimize radiation-induced toxicities, one should aim to minimize the dose by a high gradient falloff.

Studies show that a fractionated stereotactic radiotherapy regimen administered to patients with brain metastases significantly reduces the risks of symptomatic radiation-induced necrosis. Andruska et al. [16] reported a significant increase in the rate of necrosis in patients treated with radiosurgery, using V_25_ (the volume of brain tissue receiving the 25 Gy dose) and V_30_, respectively, as indicators of the success of the fractionation regimen. Cases where volumes of healthy brain tissue exceeded V_25_ ≥ 16 cm^3^ and V_30_ ≥ 10 cm^3^ were associated with a significantly higher risk of radionecrosis. In a study on 39 patients treated with 25–30 Gy in 5 fractions, Rogers et al. [17] showed no significant toxicity when the volume of normal brain tissue defined as brain-GTV did not exceed 20 cm^3^. Another study, conducted by Al-Omair et al. [18], reported favorable results using fractionated stereotactic radiotherapy for patients with brain metastases, with an average administered dose of 30 Gy/5 fr both in patients who previously underwent WBRT and as first treatment. Of 30 patients treated previously with WBRT, 3 presented radionecrosis. Kim et al. [19] reported better results using a fractionated regimen (36 Gy/6 fr) with similar overall survival to patients treated with SRS, with the benefit of a lower risk of toxicities. A recent study by Myrehaug et al. [20], on a cohort of 220 patients with 334 brain metastases, performed a 10.8-month (average) follow-up of patients treated with a fractionation scheme of 30 Gy in 5 fractions and showed symptomatic radionecrosis in only 9.5% of cases, with this being considered an acceptable risk. Treatment of brain metastases with CyberKnife using hypofractionated radiotherapy regimens also proved successful in a study by Mengue et al. [21] that evaluated a cohort of 389 patients and revealed a rate of only 5% of symptomatic necrosis.

In a cohort of 120 patients with brain metastases, Putz et al. [22] reported a necrosis rate of only 3.4% in patients treated with a fractionated regimen, while patients treated with one session of radiosurgery had reported a radionecrosis rate of 14.8%. For radiosurgery treatments, the indicator used for normal brain tissue assessment was the V_10Gy_, and this was aimed to be kept below 10 cm^3^.

Optimal fractionated radiotherapy regimens are, however, difficult to define. A longer follow-up of patients and more clinical experience is needed to establish an optimal fractionation regimen, including the choice of prescription isodose line and the assessment of toxicity rates. Meanwhile, administration of 30 Gy in 5 sessions has been shown to be effective and safe. In our institution, V_20_ of normal brain tissue is used as the main prognostic factor for the incidence of radionecrosis and is kept below 10 cm^3^, with an average of 6.1 cm^3^ for FFF optimizations and 6.28 cm^3^ for FF optimizations (+2.9%). This limit is increased for tumors with a larger volume of the PTV (>10 cm^3^) or for the treatment of multiple brain metastases using a single isocenter technique. For a single fraction, FFF beams reduced the mean dose to healthy brain tissue [23], though the differences are difficult to quantify given the very small doses administered to small tumor volumes. Our study showed no significant differences in mean dose to healthy brain tissue (1.28 Gy for FFF plans and 1.3 Gy for FF plans) due to the same factors. These values are in agreement with the dosimetric results published by Fiorentino et al. for fSRT [24]. None of the 18 patients treated with this fractionated regimen showed signs of radiation necrosis during treatment or the 3 months follow-up.

A monitor unit is a measure of machine output for linear accelerators and depends on tumor size, treatment technique, prescribed dose and plan complexity. A large number of monitor units delivered during radiotherapy is associated with increased treatment time. Thus, our study also aimed to compare the number of MUs with data reported by others. Rieber et al. [25], using Elekta Versa HD equipped with an Agility MLC system (the same configuration as the one involved in this study), reported a slightly reduced number of MUs for SRS using the FFF mode (−1.6%) compared to the FF mode, comparable with our results that showed a difference of −2% when FFF beams were employed. For SRS treatments, Stieler et al. [26] used the same device as Rieber [25] and Monaco 3.3 TPS, with the aim to compare FFF vs. FF VMAT treatment plans for single and multiple brain metastases. For single lesions, a slight decrease of MUs was reported when FFF beams were employed (−1.2%). On the other hand, for multiple brain metastases, FFF beams showed a higher number of MUs (+8%). In their study, Prendergast et al. [27] included different dose prescription for the stereotactic treatment of the central nervous system (CNS) and for more than half of the treatment plans (14 out of 27), reported a number of MUs < 2000 for the fractionated regimens, in agreement with our results (1261.2 MUs, mean value). SRS treatments showed a higher number of MUs (≥4000 MU) and prolonged BOT.

The number of MUs is directly correlated with overall treatment time. The total treatment time for stereotactic treatments is prolonged in comparison to standard treatments due to the higher number of MUs and more complex treatment plans which involve non-coplanar beams. It incorporates an important physical parameter, the beam on time, which is the actual time the radiation beam is on during a fraction [27]. A prolonged treatment time is associated with the probability of intrafractional errors due to patient movement; thus, a reduced BOT is a key characteristic of SRS and fSRT treatments where a very high precision in treatment delivery is required. Prendergast et al. [27] reported a mean BOT of 81 sec for all fractionation regimens included in their study, similar to our average BOT of 90.3 s. Studies that used FFF beams reported a dramatically reduced BOT compared with flattened beams, in agreement with our data that show a significant difference in BOT between the two beam settings (*p* ≤ 0.001). Using the Elekta Versa HD with the same MLC system, Stieler et al. [26] and Rieber et al. [25] reported a 50% and 57.9% BOT reduction, respectively, for FFF compared to FF techniques for stereotactic treatments. Dzierma et al. [28] observed a significant reduction in delivery times when FFF beams were used (50%); however, from a dosimetric perspective, the differences between FFF and FF plans can be considered clinically insignificant.

Another important parameter for fSRT treatment evaluation is the conformity index. The CI cannot be considered as only a physical parameter because it is also correlated with the dosimetric aspects of a treatment plan in terms of coverage of the target volume. In the ideal situation, the value of CI is 1 and indicates a perfect coverage of the target volume with the prescribed dose. Our study reported an average value of the CI for the FFF mode of 0.72 and 0.7 for the FF mode, which indicates an underdosage of the PTVs according to RTOG recommendations. The expected value of CI was less than 1 for all 18 cases, but it was in agreement with the requirement whereby 95% of prescribed doses should cover at least 80% of the PTV. Similar results were reported in the literature by Sarkar et al. [29], who evaluated the CI value of PTV for the Agility 6 MV FFF model, which included both single fraction and five fraction regimens, and reported a value of 0.6. Using FFF and FF beams for SRS brain metastases, Rieber et al. [25] obtained a mean CI = 0.685 for FFF plans and 0.694 for FF plans. Lai et al. [23] evaluated the dosimetric superiority of FFF compared to FF beams in VMAT plans and reported no significant differences in conformity for single fraction treatments. These results are similar to our study in which the CI value showed no statistically significant difference (*p* = 0.28).

Gasic et al. [30] conducted a study on several anatomical locations, including a cohort of patients with brain metastases that did not reveal differences between treatment plans made with FFF vs. FF beams. From a dosimetric perspective, Stieler et al. [26] reported similar treatment plans and delivery, with no clinically important differences observed when using FFF beams. The dosimetric evaluation of our plans showed similar results in terms of coverage of GTV and CTV, hotspot and CI, with no significant differences between the two optimizations. A statistically significant *p*-value of 0.036 was reported for PTV coverage, mainly due to the FFF beam characteristic (central peak) which brings the key benefit of achieving a high dose falloff at target edges. For FF optimizations, mean PTV coverage is in the 82.9–98.3% range, with the lower values being correlated with cases where sensitive OARs are near the target volume.

The high dose falloff is highlighted by the gradient index values which are comparable between FFF and FF optimizations (*p* = 0.4). The favorable gradient falloff of this technique is correlated with good clinical prognoses in terms of toxicities. In their study employing Gamma Knife SRS, Greto et al. [31] showed a notable overall survival at 1 year (74%) and only 1 case of radionecrosis without neurological symptoms that was associated with the benefit of high dose falloff offered by the irradiation technique. At the same time, fSRT provides lower toxicity rates due to the fractionation regimen which allows normal brain tissue recovery time between fractions. As reported in our study, all 18 patients completed treatment without toxicity-caused interruption or early signs of radiation necrosis.

The findings of this work must be seen in light of some potential limitations. The primary limitation is the reduced cohort size (18 patients) which can impact on data generalizability. The lack of comprehensive toxicity reporting due to the short follow-up period (3 months) is another shortcoming of our study. A long-term follow-up study reporting on both treatment outcome and normal tissue effects is required.

## 5. Conclusions

This study compared the dosimetric results for fSRT treatments with a prescribed dose of 30 Gy in 5 fractions optimized for both FFF beams and FF beams. The cohort included 18 patients with brain metastases treated with FFF beams, as per institutional protocol. From a dosimetric viewpoint, there were no statistically significant differences between the two optimizations: tumor volume coverage was achieved according to dosimetric requirements, and doses to organs at risk were similar for both beam settings. The dosimetric results obtained by our study encourage the implementation of fSRT with standard flattened beams in centers where FFF linacs are not available. The main advantage of FFF beams is the beam on time (delivery time), which is significantly reduced compared to FF beams, leading to better patient comfort during treatment.

## Figures and Tables

**Table 1:** Baseline characteristics of patients.

Patient Characteristics	Number (%)/Mean ± Std Dev	
Number of patients	18	
Sex		
Male	9 (50%)	
Female	9 (50%)	
Age		
Mean age	65.8 ± 9.6 years	
Mean age male	70.1 ± 5.4 years	
Mean age female	61.4 ± 11.1 years	
Primary lesion		
Lung	11 (61.1%)	
Breast	4 (22.2%)	
Renal	2 (11.1%)	
Melanoma	1 (5.6%)	

**Table 2:** Tumor volume and beam set-up.

Patient	Number of PTVs	Volume of PTV (cc)	Number of Fields	Couch Rotation
1	2	5.45	4	315/0/45/90
2	1	6.08	3	0/90
3	1	5.59	4	45/0/315/270
4	2	5.39	3	0/45/90
5	2	2.96	3	315/45/90
6	1	4.47	2	0/90
7	1	6.11	2	0/60
8	2	7.52	4	0/45/315
9	1	4.49	3	35/325/270
10	1	6.96	3	0/60/300
11	1	2.39	3	35/325/270
12	1	2.49	3	0/315/270
13	2	6.15	2	0/90
14	1	9.77	2	0/45
15	1	2.34	3	0/45/270
16	1	5.39	3	0/270
17	1	5.89	3	0/45/90
18	1	4.40	3	0/45/90

**Table 3:** Dose constraints for target volumes and organs at risk.

Target Volume	Hotspot	Coverage
GTV	$D_{min}\geq 120\%$ $D_{max}$ ≤ 140%	$D_{100\%}\geq 100\%$
CTV	$D_{max}$ ≤ 140%	$D_{98\%}\geq 98\%$
PTV	$D_{max}$ ≤ 140% $D_{105\%}$ to be kept inside PTV	$D_{95\%}\geq 80\text{–}100\%$
OARs	Maximum dose	Volumetric constraints
Brainstem	$D_{max}\leq 20\;Gy$	$V_{20\;Gy}\leq 1\text{–}10\;cc$
Optic nerves	$D_{max}\leq 12\text{–}15\;Gy$	
Optic chiasma	$D_{max}\leq 12\text{–}15\;Gy$	
Lens	$D_{max}\leq 8\;Gy$	
Cochlea	$D_{max}\leq 10\;Gy$	
Normal brain tissue		$D_{mean}\leq 8\;Gy\;V_{20\;Gy}\leq 10\;cc$

**Table 4:** Physical dosimetric parameters.

Patient	PTV Volume (cc)	MU		CI		DGI		Estimated Delivery Time According to TPS (s)		Beam on Time (s)	
		FFF	FF	FFF	FF	FFF	FF	FFF	FF	FFF	FF
1	5.45	1776.7	1758.3	0.45	0.45	70.9	68.7	178.46	176.4	126	265
2	6.08	1187	1199.5	0.85	0.82	86.4	85.3	115.21	114.63	66	172
3	5.59	1242.8	1364.7	0.77	0.79	55.7	62.3	159.65	169.46	126	210
4	5.39	1622.1	1716.3	0.41	0.41	73.5	70.3	164.2	173.08	78	260
5	2.96	1672.1	1705.4	0.4	0.4	62.2	62.8	161.47	165.71	102	249
6	4.47	1219.9	1271.4	0.88	0.89	85.8	85.3	123.1	126	90	189
7	6.11	1213.8	1216.1	0.85	0.85	82.9	82.4	117.86	114.5	78	172
8	7.52	1286.5	1252	0.41	0.44	66.6	65.2	191	187.6	102	281
9	4.49	1318	1267.68	0.85	0.84	84.6	84.2	153	145.8	126	219
10	6.96	927.6	733.5	0.91	0.6	63.6	45.1	107.8	94.2	90	141
11	2.39	1332.3	1341.5	0.8	0.8	83.5	83.4	129.5	133.12	84	200
12	2.49	1219.5	1267.1	0.75	0.73	85.1	83.8	120.87	124.22	78	200
13	6.15	1328.9	1284.7	0.4	0.4	65.0	63.9	135.6	132.2	90	198
14	9.77	773.1	863	0.82	0.82	58.6	59.1	74.61	87	48	131
15	2.34	1034.71	1141.4	0.89	0.89	90.5	90.4	120	127.3	90	194
16	5.39	1062.44	1135.9	0.89	0.86	82.4	81.7	154	162.71	78	244
17	5.89	1305.28	1212.2	0.83	0.8	65.5	62.9	132.94	120.9	84	181
18	4.40	1178.09	1390.58	0.85	0.87	76.8	77.1	127.32	141.07	90	221
Average	5.21	1261.2	1284.5	0.72	0.7	74.4	73	137	138.7	90.3	207
*p*-value		0.29		0.28		0.4		0.4		≤0.001	

**Table 5:** Dosimetric evaluation for PTVs and OARs.

Patient	No. of PTVs	PTV Vol. (cc)	GTV		CTV		PTV				Normal Brain			
			Coverage (%)		Coverage (%)		Coverage (%)		Hotspot (Gy)		V_20_ (cc)		Mean Dose (Gy)	
			FFF	FF	FFF	FF	FFF	FF	FFF	FF	FFF	FF	FFF	FF
1	2	5.45	100	100	100	100	94.35	93.3	44.12	44.23	5.10	5.20	1.84	1.88
2	1	6.08	100	100	100	100	85.23	82.98	44.46	44.01	2.50	2.48	0.90	0.79
3	1	5.59	99.36	99.41	97.13	97.34	94.72	95.08	40.80	41.82	4.20	3.62	1.27	1.20
4	2	5.39	100	100	99.32	99.26	95.37	94.50	42.33	41.09	4.60	4.83	1.10	1.14
5	2	2.96	100	100	98.26	96.28	91.67	88.80	40.97	41.47	4.94	4.60	1.52	1.51
6	1	4.47	100	100	99.97	99.97	97.22	97.03	41.50	41.80	8.16	8.28	1.30	1.32
7	1	6.11	100	100	100	100	96.09	96.10	41.60	42.03	1.95	1.97	1.06	1.09
8	2	7.52	100	100	97.73	99.10	95.66	95.39	41.09	41.39	6.47	6.63	1.95	2.02
9	1	4.49	100	100	100	100	95.57	95.23	42.31	41.40	8.55	8.68	1.07	1.11
10	1	6.96	100	99.66	99.90	96.00	99.60	90.67	41.19	36.64	7.42	11.03	1.44	1.70
11	1	2.39	100	100	98.10	97.80	92.69	94.47	41.67	42.86	5.53	5.51	0.97	0.98
12	1	2.49	100	100	100	100	89.13	88.89	44.50	45.25	1.24	1.26	0.85	0.88
13	2	6.15	100	100	99.99	99.94	98.27	98.30	38.31	38.77	9.71	9.94	1.97	2.03
14	1	9.77	100	100	98.32	92.33	93.92	93.65	38.13	32.54	10.00	9.80	1.99	1.98
15	1	2.34	100	100	100	100	97.56	97.52	39.59	39.53	3.27	3.27	0.62	0.63
16	1	5.39	100	100	99.00	98.31	89.34	86.22	34.23	41.55	8.37	8.21	1.14	1.13
17	1	5.89	99.92	99.76	98.42	96.44	87.01	83.42	37.70	36.03	10.00	10.00	1.13	0.99
18	1	4.4	100	100	100	100	98.63	97.84	35.83	36.00	7.87	7.67	0.95	0.93
Mean		5.21	99.96	99.94	99.23	98.49	94.00	92.74	40.57	40.47	6.10	6.28	1.28	1.30
*p*-value			0.246		0.088		0.036		0.864		0.419		0.51	

## Data Availability

The data that support the findings of this study are available from the corresponding author upon reasonable request.
